# Whole-genome sequencing uncovers diverse genetic causes and phenotypic signatures in infantile nystagmus and albinism

**DOI:** 10.1038/s41525-026-00580-1

**Published:** 2026-05-20

**Authors:** Mahmoud R. Fassad, Pradeep C. Vasudevan, Julian Barwell, Gail DE Maconachie, Savita Madhusudhan, David Hunt, Irene Gottlob, Mariya Moosajee, Omar A. Mahroo, Andrew R. Webster, Michel Michaelides, Mervyn G. Thomas

**Affiliations:** 1 The University of Leicester Ulverscroft Eye Unit, Robert Kilpatrick Clinical Sciences Building, School of Psychology and Vision Sciences, Leicester, UK; 2 Department of Clinical Genetics, University Hospitals of Leicester NHS Trust, Leicester Royal Infirmary, Leicester, UK; 3 Human Genetics Department, Medical Research Institute, Alexandria University, Alexandria, Egypt; 4 School of Allied Health Professions, Nursing and Midwifery, The University of Sheffield, Yorkshire, UK; 5 St. Paul’s Eye Unit, Royal Liverpool University Hospital, Prescot Street, Liverpool, UK; 6 Department of Eye and Vision Sciences, University of Liverpool, William Henry Duncan Building, 6 West Derby Street, Liverpool, UK; 7 Wessex Clinical Genetics Service, Princess Anne Hospital, University Hospital Southampton NHS Trust, Southampton, UK; 8 Department of Neurology, Cooper University Hospital, Camden, NJ USA; 9 UCL Institute of Ophthalmology, University College London, London, UK; 10 NIHR Biomedical Research Centre at Moorfields Eye Hospital and the UCL Institute of Ophthalmology, London, UK; 11 Department of Ophthalmology, University Hospitals of Leicester, Leicester Royal Infirmary, Leicester, UK

**Keywords:** Diseases, Genetics, Medical research

## Abstract

We combined deep phenotype data and whole-genome sequencing results from the UK 100,000 Genomes Project (100KGP) to characterize the genetic spectrum of infantile nystagmus and albinism, and to explore genotype–phenotype correlations in this cohort. Participants were enrolled in the study based on the clinical codes for albinism or infantile nystagmus. Whole genome sequencing data was retrieved and variants in known nystagmus/albinism genes (PanelApp R39 panel) were analysed alongside genome-wide findings. We ascertained 473 affected individuals (388 families). Positive genetic findings were obtained in 218 participants (46%), including 176 (37%) with definitive diagnoses. Pathogenic variants were found in 16 of 38 panel genes, most commonly in *TYR* (56 families) and *OCA2* (21 families). Recurrent variants (24% of 103 different variants) were identified in six genes. An additional 36 off-panel genes accounted for diagnoses in 45 families, including four dual genetic diagnoses in three families. Phenotypically, refractive errors and foveal hypoplasia-related diagnoses were significantly enriched (odds ratio ~2.8 for refractive disorders). Strabismus and neurodevelopmental features were also over-represented in the cohort. We show that whole-genome sequencing provided a high diagnostic yield in infantile nystagmus/albinism and uncovered a broad allelic spectrum. Integrating comprehensive phenotype data identified distinct genotype–phenotype relationships.

## Introduction

Infantile nystagmus (IN, also known as congenital nystagmus) is a broad clinical term referring to disorders characterised by involuntary, rhythmic oscillations of the eyes that typically present within the first six months of life^1^^,2^. It has an estimated prevalence of approximately 6.1 per 10,000 live births, depending on the population studied^3,4^. IN is a genetically and phenotypically heterogeneous condition, often associated with developmental abnormalities of the retina, optic nerve, or brain. While most cases are due to genetic causes - including albinism, inherited retinal disorders, or idiopathic IN - non-genetic aetiologies must be carefully excluded. These include acquired structural brain lesions, such as space-occupying tumours or intracranial malformations, particularly in cases with late-onset or asymmetric findings^5^. Early and accurate classification of IN is therefore crucial, not only to guide appropriate investigations and genetic testing, but also to identify syndromic associations that may have broader clinical implications. IN frequently co-occurs with conditions affecting the fovea or optic pathway, most notably albinism. Albinism refers to a group of genetic disorders of melanin biosynthesis characterised by hypopigmentation of the eyes, and often the skin and hair^1^. In albinism, absent or reduced pigmentation leads to structural eye abnormalities such as foveal hypoplasia and optic nerve misrouting. The genetic architecture of albinism is also highly heterogeneous. There are multiple oculocutaneous albinism (OCA) subtypes, primarily caused by autosomal recessive variants in genes such as *TYR* (OCA1), *OCA2* (OCA2), and others. In European populations, *TYR* and *OCA2* together account for roughly 70% of OCA cases ^3^. X-linked ocular albinism (OA1) is caused by *GPR143* variants and constitutes around 7% of albinism cases. The remaining cases are explained by less common genes including those associated with Hermansky–Pudlak syndrome and other rare forms. Because infantile nystagmus can be a unifying presentation, patients are often initially labelled under broad terms (e.g., “nystagmus” or “albinism”) even though the underlying genotypes span dozens of genes. Traditional diagnostic approaches, such as targeted gene panels, may focus on the most common genes for a presumed diagnosis (for example, an “albinism” gene panel) and could miss atypical or syndromic causes.

Advances in genomic medicine, particularly a disease-agnostic analysis of whole-genome sequencing (WGS) data, enables a more comprehensive investigation of patients with genetically heterogeneous conditions. WGS captures sequence information across the entire genome, including coding regions, splice sites, and non-coding regulatory elements. In clinical practice, in silico targeted panel analysis of WGS data has proven both sensitive and time-efficient, enabling phenotype-driven variant identification while maintaining the flexibility to investigate off-panel genes when initial analysis is non-diagnostic^6,7^. Furthermore, large initiatives like the UK’s 100,000 Genomes Project (100KGP) provide an opportunity to study genotype–phenotype relationships at scale. Early studies from the 100KGP and other WGS cohorts have reported diagnostic yields around 25–40% across diverse rare diseases, but certain ophthalmic conditions may achieve higher yields with careful phenotypic selection^8^. A recent review of genetic testing in infantile nystagmus syndrome found molecular diagnosis rates of 35–60% using targeted panels^9^, which rose to ~80% when testing was guided by specific clinical findings (and up to 88% with a positive family history) ^10^. Moreover, genetic results can refine the clinical diagnosis in up to 30% of patients, highlighting the importance of genomic analysis for appropriate patient classification^11^.

In this study, we utilised the nationwide 100KGP dataset^12^ to investigate infants and children with nystagmus and albinism phenotypes. Our aims were to (1) determine the diagnostic yield of in silico panel analysis of WGS data in this cohort, (2) characterize the spectrum of genetic variants, including novel and recurrent mutations, and (3) explore genotype–phenotype correlations by integrating detailed clinical data (Human Phenotype Ontology terms, ICD-10 codes, and rare disease categories). We hypothesized that a combined analysis of genomic and phenotypic data would not only improve diagnostic discovery but also reveal patterns (e.g., specific co-occurring features) that could help clinicians distinguish among genetic causes of infantile nystagmus and albinism.

## Results

### Demographic characteristics of the phenotype cohort

We identified 441 affected participants from 388 unrelated families via the phenotype-based search. In total, these families included 473 affected individuals (269 males and 204 females). Of these, 32 individuals did not have any of the target phenotypic terms recorded in their clinical documentation. However, they were included because they belonged to families in which one or more other members were identified based on the presence of a relevant phenotype (e.g., nystagmus or albinism), and were labelled as affected within the family context. These additional participants often shared the same variants as their relatives but lacked explicit phenotypic coding themselves. Over half of the families (204/388, 53%) were trio recruits (proband and both parents), and 53 families (14%) were singletons (**Supplementary Information -** Fig. S1). Ethnicity data was reported in 331 families (85%). 79% of families with known ethnicity (263/331) were of White background (**Supplementary Information -** Fig. S2).

### Clinical characteristics of the phenotype cohort

HPO-derived clinical data were available for 465 of the 473 participants (98%). Approximately two-thirds of the participants (312/473,66%) had one or more of the HPO terms of interest including 27 with ‘Albinism’, few (less than six) with ‘Partial albinism’, 41 with ‘Ocular albinism’, 194 with ‘Congenital nystagmus’ (including descendant terms), and 68 with ‘Aplasia/hypoplasia of the fovea’ (including descendant terms). Notably, 293 (94%) of these 312 participants had only one of the HPO terms of interest recorded, rather than multiple terms that often co-occur. For example, “Albinism” and “Ocular albinism” were reported together in few (less than six) individuals, and similarly, “Congenital nystagmus” plus a foveal hypoplasia term found in few participants.

**Fig. 1:**
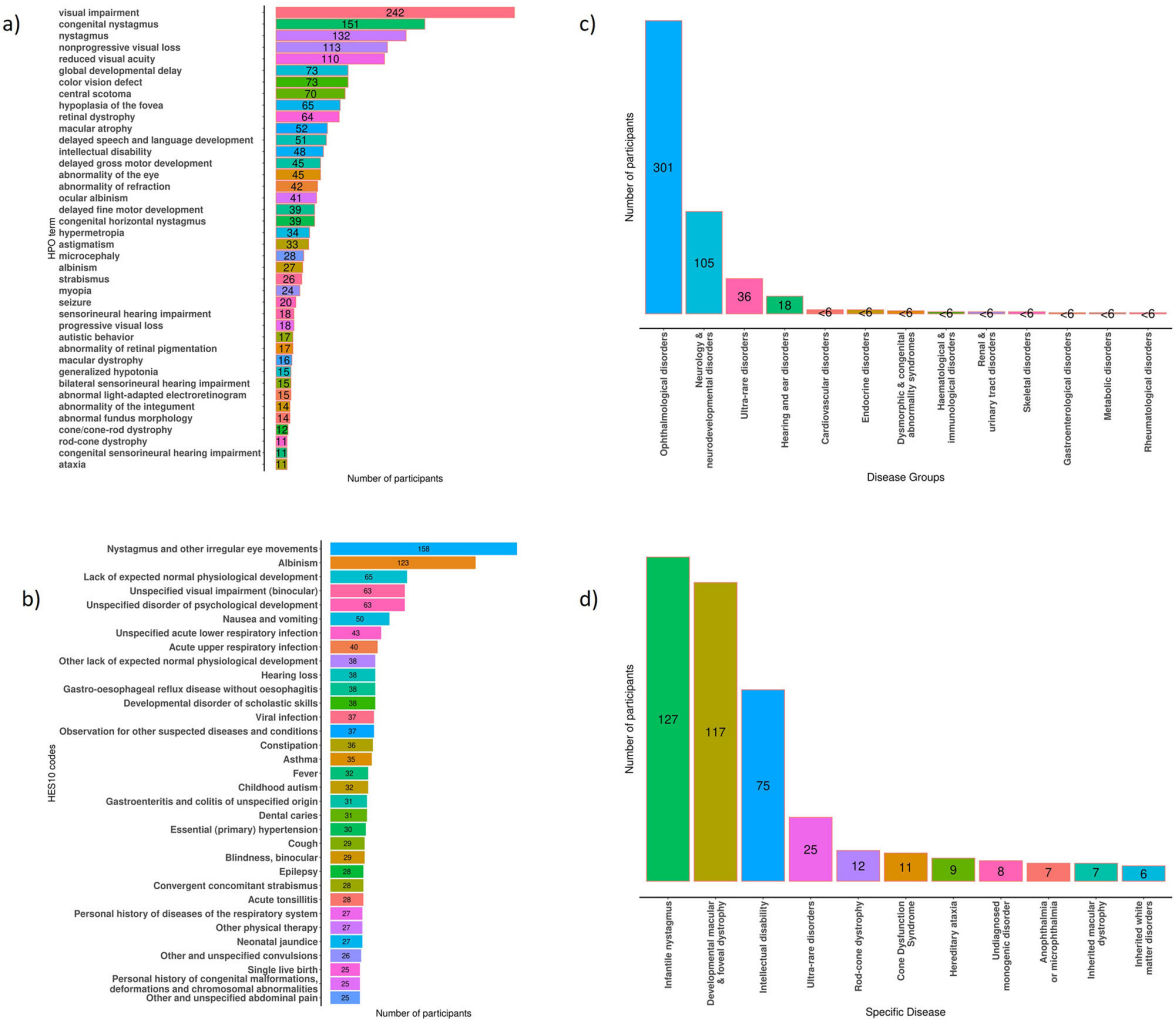
Clinical characteristics of the studied cohort. **a** Bar chart of the frequency of the most common HPO terms used to describe the clinical phenotypes of the participants. In total, there were 697 unique HPO terms used. The most frequent HPO term reported was Visual impairment followed by congenital nystagmus. **b** Bar chart of the frequency of the most common ICD-10 terms retrieved from HES reports of the participants. In total, there were 1846 unique terms retrieved. The most frequent terms were nystagmus and other irregular eye movement (H55) and albinism (E70.3). **c** Bar chart of the frequency of the GE disease group categories of the participants showing that most of the participants were included under ophthalmological disorders category. **d** Bar chart of the frequency of the most common GE specific disease categories of the participants showing that the most frequent specific diseases were infantile nystagmus, developmental macular and foveal dystrophy and intellectual disability. The colors of the bars represents different terms/diseases.

Referring clinicians used a wide array of HPO terms to describe the cohort. In total, 697 unique HPO terms were used (Supplementary Data1). There were 3052 HPO term entries for 465 participants, ranging from one to 28 terms per participant. On average, about six HPO terms were used per participant. The most frequently used HPO terms were eye-related (descendants of ‘Abnormality of the eye’). The most common HPO term was ‘Visual impairment’ used in 242 participants (52%). “Congenital nystagmus” (alone without descendants), was used in 151 participants (32%). Descendants of the ‘Neurodevelopmental abnormality’ were frequently reported including ‘Global developmental delay’ in 73 participants (16%), ‘Delayed speech and language development’ in 51 participants (11%) and ‘Intellectual disability’ in 48 participants (10%) (Fig. 1a).

Health episode statistics (HES) records (inpatient and outpatient encounters) were available for 367 of the 473 participants (78%). Of these, 123 (34%) had Albinism (ICD-10 code E70.3) documented in their HES data. The most frequently reported ICD-10 code was Nystagmus (H55), recorded in 158 participants (43%) (Fig. 1b, Supplementary Data2).

GE rare diseases codes provided an additional perspective on diagnoses. The rare diseases coding is hierarchical (group, subgroup, specific disease). In our cohort, 127 participants (27%) had ‘Infantile nystagmus’ listed as their specific rare disease. Few participants (less than six) had more than one different disease group codes (indicating a complex or overlapping diagnostic labelling) and the remaining 469 participants each had a single rare disease group. The top three disease groups categories were Ophthalmological disorders, Neurology and neurodevelopmental disorders and Ultra-rare disorders (Fig. 1c, Supplementary Data 3). After “Infantile nystagmus”, the next specific rare disease code was ‘Developmental macular and foveal dystrophy’, present in 117 participants (25%). ‘Intellectual disability’ was the third most frequent, in 75 participants (16%) (Fig. 1d, Supplementary Data 4).

The overlap between the three coding systems (HPO, ICD-10, rare disease) was very limited. Only few participants (less than six) had terms of all three systems ( ≥ 1 HPO terms of interest, an ICD-10 code for albinism, and a rare disease code for infantile nystagmus). In contrast, 198 of the 441phenotype-identified participants (45%) had one or more of HPO terms of interest without any ICD-10 or rare disease code. Combining data from all three coding systems, 133 participants (28%) had documented albinism phenotype and 189 participants (40%) had documented abnormal retinal phenotype. Isolated nystagmus with no other features suggesting syndromic presentation was reported in104 participants (22%).

### Enriched clinical features in the cohort

**Fig. 2:**
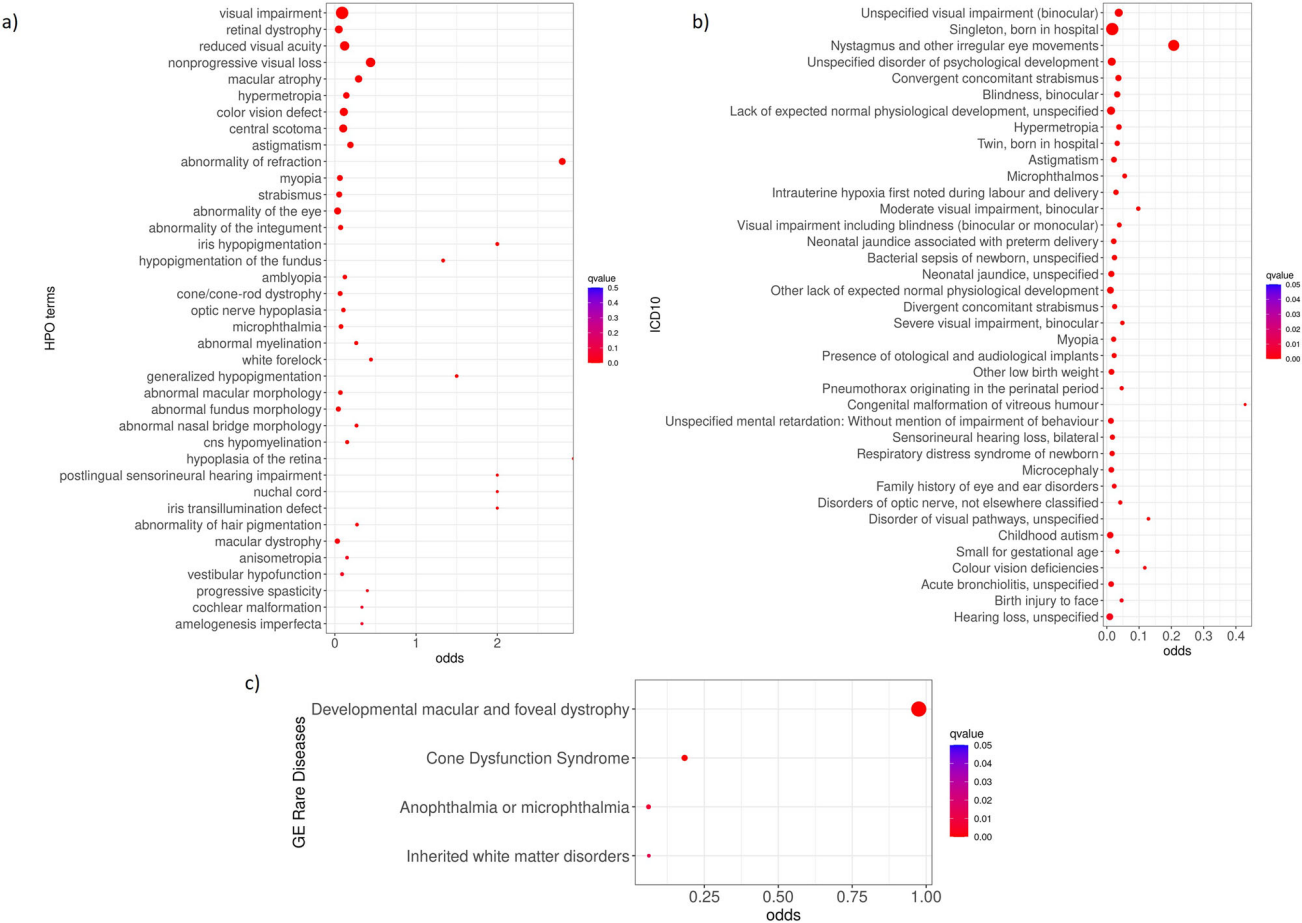
Phenotype enrichment within the infantile nystagmus/albinism cohort. **a** Dot plot of the 38 HPO terms enriched within the cohort compared to the rare diseases cohort of the 100KGP. **b** Dot plot of the most significantly enriched ICD-10 terms within the cohort (those with the lowest q values). In total, there were 85 significantly enriched ICD-10 terms. Vision impairment and nystagmus were among the most significantly enriched terms within the cohort. **c** Dot plot of the enriched GE specific diseases reported in the cohort. Developmental macular and foveal dystrophy was the most significantly enriched specific disease code with about half of the rare diseases cohort participants in the 100KGP with this disease code included in the current infantile nystagmus/albinism cohort. The size of the dot represents the number of participants with each term/disease, the color of the dot represents the q value (corrected *p* value for multiple testing). The *x* axis represents the odds of a participant (in the rare diseases cohort of the 100KGP) with each of the terms/diseases to be included in the infantile nystagmus/albinism cohort.

We performed an enrichment analysis of clinical features in our phenotype cohort compared to all 100KGP rare disease participants. HPO enrichment (excluding the HPO terms used for cohort selection) identified 38 significantly over-represented terms. Most were eye-related terms. “Abnormality of refraction” and its descendant terms (hypermetropia, astigmatism, myopia, anisometropia) were all among enriched features. Interestingly, the odds of a participant with refractive error to be included in this nystagmus/albinism cohort was 2.8. Nervous system-related HPO terms such as “abnormal myelination” (specifically “CNS hypomyelination”) were also enriched. Other extra-ocular terms enriched in our cohort included “postlingual sensorineural hearing impairment”, “cochlear malformation” and “vestibular hypofunction” (Fig. 2a, Supplementary Data 5).

Enrichment analysis of HES-derived ICD-10 codes revealed 85 significantly enriched codes (excluding Albinism). Similar to the HPO results, astigmatism (H52.2), hypermetropia (H52.0) and myopia (H52.1) were all enriched ICD-10 terms. Additionally, sensorineural hearing loss and presence of otological and audiological implants were significant enriched (Fig. 2b, Supplementary Data6).

GE rare disease code enrichment identified four specific diagnoses (besides infantile nystagmus) that were over-represented in the cohort: developmental macular and foveal dystrophy, cone dysfunction syndrome, anophthalmia or microphthalmia and inherited white matter disorders (Fig. 2c, Supplementary Data 7).

### Diagnostic yield of whole genome sequencing

We classified participants with positive genetic findings as: solved, partially solved and possibly solved. “Solved” cases were those in which pathogenic/likely pathogenic variant(s) segregated appropriately and fully explained the phenotype. “Partially solved” cases had a variant(s) that explained some features but not all of the participants’ phenotype(s).“Possibly solved” cases had a strong candidate variant(s) without segregation data or those with segregating variants of uncertain significance (VUS).

Applying the R39 panel analysis, we identified causative/likely-causative variants in 159 participants from 133 unrelated families. Through the additional review of GMC reports, we found that 63 participants from 45 families had pathogenic variants in genes outside the R39 panel. In total, 218 participants (46% of 473) from 175 families had a positive genetic finding (solved, partial, or possible).

**Fig. 3:**
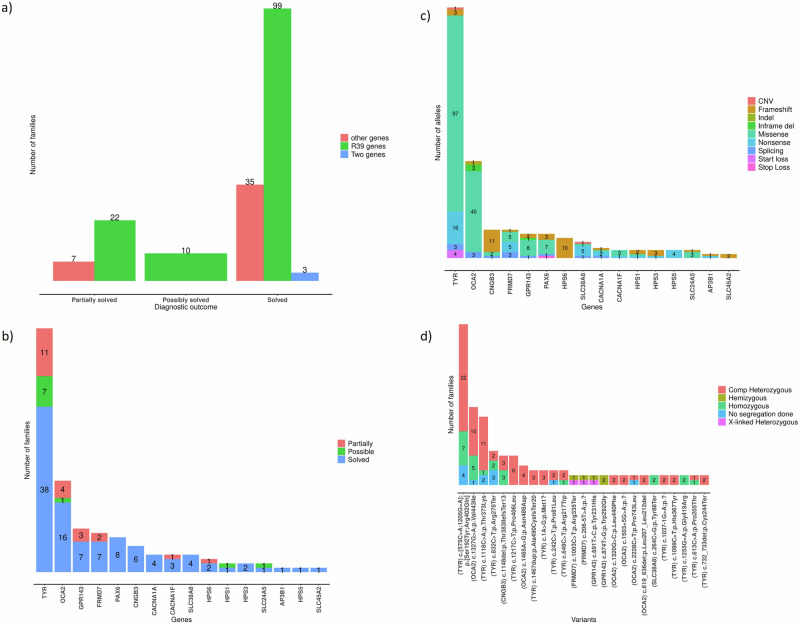
Genetic diagnostic outcome and genetic spectrum of the cohort. **a** Grouped bar chart showing the number of families solved, partially solved or possibly solved by variants in and/or off R39 panel genes. Most families had variants in genes in the R39 panel. Three families were solved with dual diagnosis with variants in gene in and off the R39 panel. **b** Stacked bar chart of the number of families solved with variants in 16 genes in the R39 panel. The most frequently mutated genes were *TYR* and *OCA2*. **c** Stacked bar chart of the number of various types of variants identified in the R39 panel genes. Most of the variants identified in *TYR* and *OCA2* were missense variants. On the other side, most of the variants identified in *CNGB3* were frameshift variants. **d** Stacked bar chart of the frequency of the most identified variants (mutant alleles) and their corresponding genotypes. The *cis*-YQ haplotype in *TYR* gene was the most frequent allele identified within the cohort.

In total, 176 participants (37% of the cohort), from 137 families were classified as *solved*. This comprised 120 participants (99 families) with variants in R39 panel genes and 52 participants (35 families) with variants in genes outside the R39 panel (Fig. 3a). Four participants from three families had a *dual diagnosis*, with pathogenic variants in both R39 gene and a different gene that together explained a complex phenotype (Fig. 3a). In 32 participants (7%) from 29 families, the identified variant(s) explained only part of the phenotypes (*partially solved*). These included 25 participants (22 families) with variants in R39 genes and 7 participants (7 families) with variants in genes outside than R39 panel (Fig. 3a). Furthermore, 10 participants were classified as *possibly solved* by variants in R39 genes (these were cases where the family was a singleton lacking segregation data, or a variant was deemed a VUS) (Fig. 3a, Supplementary Data 8).

### Genetic spectrum of albinism/congenital nystagmus cohort

We identified variants in 16 out of 38 Green/Amber genes on the R39 panel. The most frequently mutated genes were the autosomal recessive *TYR* (56 families, 62 participants) and *OCA2* (21 families, 24 participants). Variants were identified in other autosomal recessive genes: *CNGB3* (6 families, 7 participants), *SLC38A8* (4 families, 4 participants), *HPS6* (5 participants from 2 families), *HPS1* (2 families, 2 participants), *HPS3* (2 families, 2 participants) and *SLC24A5* (2 families, 2 participants). Additionally, we found variants in one family for each of the following genes: *AP3B1*(1 participant), *HPS5* (2 participants) and *SLC45A2* (2 participants). Among autosomal dominant genes, *PAX6* had pathogenic variants in 8 families (12 participants) and *CACNA1A* in 4 families (5 participants). X-linked genes with disease-causing variants included *GPR143* in 10 families (12 participants), *FRMD7* in 9 families (14 participants), and *CACNA1F* in 4 families (4 participants) (Fig. 3b).

Collectively, 271 disease-causing alleles were identified in 159 participants. This total consists of 224 autosomal recessive alleles (in 112 participants), 17 autosomal dominant alleles (17 participants) and 30 X-linked alleles (30 participants). The most common variant type was missense, comprising 168/271 alleles (62%) and these missense changes were distributed across 11 different genes. Truncating variants were less common: frameshift variants accounted for 41/271 (15%) alleles (in 13 genes) and nonsense variants 36/271 (13%) alleles (in 9 genes) (Fig. 3c).

We identified 103 distinct pathogenic variants across 16 R39 panel genes. Most (78 variants,76%), were unique to single families (private variants), while 25 variants (24%) recurred in two or more unrelated families. The recurrent variants were found in *TYR, OCA2, CNGB3, FRMD7, GPR143* and *SLC38A8* genes (Fig. 3d, Supplementary Data 8). The most frequently observed disease-causing variant was the *TYR* “*cis*-YQ” p.[Ser192Tyr;Arg402Gln] haplotype. It was identified in homozygous state in 7 families and in compound heterozygous state with another pathogenic/likely pathogenic variant in 22 families. In 4 families, although *cis*-YQ haplotype was identified along with another pathogenic/likely pathogenic variant in the affected individuals, no segregation studies were done to confirm that the variants were in *trans*. As most recruited families were White Caucasians, the majority of recurrent variants were seen in White participants. However, some variants were shared across multiple ethnic groups – for example, *TYR* p.Arg278Ter was identified in both White Caucasian and South Asian families. Other variants were confined to non-White families such as *TYR* p.Gly419Arg and *SLC38A8* p.Tyr88Ter, which were only identified in South Asian families.

Through the GMC laboratories feedback, we also identified pathogenic variants in 36 genes outside the R39 panel. The most recurrent of these were genes associated with Waardenburg syndrome. Causal variants in *SOX10* were identified in five families (fully solving four and partially solving one). *MITF* disease-causing variants fully solved four families. Furthermore, a previously reported *PAX3* variant was also identified to partially contribute to the phenotype in one family. Other off-panel diagnoses included achromatopsia due to *CNGA3* variants (in two families) and a congenital disorder of glycosylation due to *SRD5A3* (in two families) (Supplementary Data 9).

Among the 36 off-panel genes identified, neurodevelopmental disorder-associated genes represented a substantial proportion. These included genes associated with well-characterized syndromes such as *ARID1B, TUBB4A* and *SYNGAP1* (Supplementary Data 9). Collectively, neurodevelopmental disorder genes accounted for about two thirds of the off-panel diagnoses. Nystagmus in these conditions likely reflects either cortical visual processing abnormalities or broader impacts of neurodevelopment on oculomotor control pathways.

### Genotype/phenotype correlations

We first assessed phenotype enrichment within specific genetic subgroups. We examined participants carrying pathogenic variants in *TYR*, *OCA2*, *GPR143*, *FRMD7*, and *PAX6*, looking for HPO terms enriched relative to the rest of the IN/albinism cohort. HPO enrichment analysis showed that anisometropia was the only HPO term significantly enriched in participants with variants in *PAX6*.

**Fig. 4:**
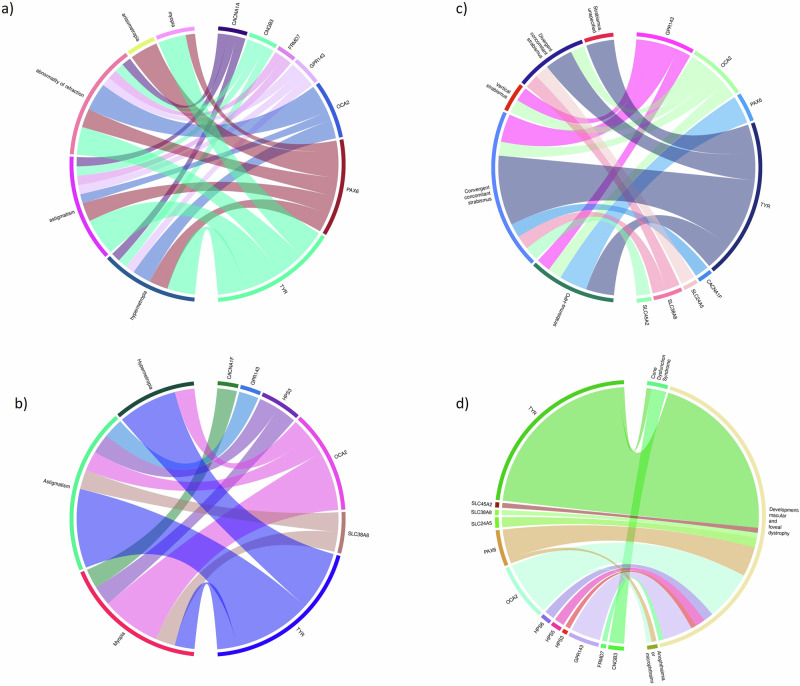
Genotype/phenotype correlations within the cohort. **a** Circos diagram showing the associations between refractive errors on the left side of the diagram (based on the reported HPO terms) and the corresponding mutated genes on the right side of the diagram. Anisometropia was exclusively reported in participants with *PAX6* variants. **b** Circos diagram of the association between ICD-10 terms of refractive errors (based on the HES data) and the corresponding mutated genes. **c** Circos diagram of the association between various types of strabismus reported in the HES and HPO data and the corresponding mutated gene. The most common types reported were the convergent and divergent concomitant strabismus. **d** Circos diagram of the association between the GE specific diseases and the mutated genes showing that developmental macular and foveal hypoplasia was the most frequently reported disease across all mutated genes. Cone dysfunction syndrome was almost exclusively reported in *CNGB3* patients. Surprisingly, cone dysfunction syndrome was also reported in one participant with *TYR* variants (although this turned out to be a mislabelling error). The breadth of the links represents the number of participants with each term/disease and the mutant gene.

Second, we explored associations between specific clinical features and the mutated genes. Refractive errors, as documented by HPO or HES (ICD-10) entries, were observed in participants with variants in 11 different genes. Astigmatism had the broadest genetic association, being reported in participants with variants in 10 of those genes. In contrast, anisometropia was observed only in participants with *PAX6* variants, aligning with the enrichment result above. Participants with pathogenic variants in *TYR*, *OCA2*, and *PAX6* collectively showed a range of refractive errors including astigmatism, myopia, and hypermetropia (Fig. 4-a,b). We also examined strabismus in the cohort: in total, strabismus (of any type) was reported in participants with variants in 8 genes. The predominant types were convergent concomitant strabismus and divergent concomitant strabismus, as recorded in the HES data (Fig. 4c).

Finally, we investigated the correlations between the enriched GE diseases rare disease diagnoses and specific genotypes. The rare disease code “Developmental macular and foveal dystrophy” was the most common label across participants with various gene mutations (reflecting that many genetic causes in this cohort result in foveal underdevelopment). In contrast, “cone dysfunction syndrome” was almost exclusively associated with *CNGB3* variants (achromatopsia), with only one *TYR* case carrying that label (hospital data confirmed this is a mislabelling error). “Anophthalmia or microphthalmia” was primarily associated with *PAX6* variants, consistent with the known spectrum of eye malformations in *PAX6* haploinsufficiency and was noted in one partially solved *OCA2* case (Fig. 4d).

## Discussion

IN is a highly heterogeneous condition with a wide range of genetic and non-genetic causes. Genetic diagnosis is essential for IN as it helps better understanding of the basis of the condition and informs clinical management specially in the context of a broader syndromic disorder with systemic implications. In the current study, we used a phenotype-driven approach to ascertain patients with IN and albinism from the 100 KGP. Among 388 unrelated families of diverse ethnicities, we implemented in silico targeted gene panel analysis of WGS data to identify the genetic causes of infantile nystagmus/albinism.

We collected detailed phenotype data using three complementary coding systems (HPO terms, HES records and GE disease categories) to capture the full spectrum of clinical features. The high phenotypic heterogeneity of the cohort is very evident in the large numbers of unique HPO (697 including 68 terms present in six or more participants) and ICD-10 (1846 including 289 terms present in six or more participants) terms used to describe the clinical features of the participants. Using multiple coding frameworks also helped to mitigate gaps or inaccuracies that might arise when relying on a single system. For example, some participants had as few as one HPO term documented; in such cases, the additional information from HES records and GE disease category labels provided a more complete clinical picture. Although participants with identified acquired brain lesions causing secondary nystagmus would not meet 100KGP eligibility criteria, we did not systematically review neuroimaging for all cases. However, the high proportion of genetic diagnoses achieved (46%) and the absence of documented structural brain pathology in clinical coding systems suggest that non-genetic structural causes are unlikely to substantially impact the observed diagnostic yield.

Enrichment analysis of the clinical features confirmed previous observations: individuals with IN are more likely to have refractive errors than the general population^13^. In particular, astigmatism and hypermetropia were more frequently reported than myopia in our cohort, consistent with previous studies of refractive error profiles in IN/albinism^14,15^. Similarly, nervous system-related phenotypes were enriched, in line with a previous report that ~30% of infantile nystagmus have an underlying neurological cause^16^. This highlights the significance of brain MRI as a diagnostic modality for IN patients, especially in patients who also exhibit other neurological symptoms^17^.

Our in silico WGS panel analysis yielded a diagnostic output of 46%, which is comparable to diagnostic yields previously reported between ~35% and 60% in other panel-based NGS studies^10,11,18–20^. Such variability might be attributed to factors including the size and characteristics of the cohort, number of genes in the panel, differences in sequencing technology or data analysis workflow. The current study represents the largest cohort investigated with 441 participants compared to other cohorts ranging between 15 and 117 participants. We only based our analysis on coding variants or those predicted to affect splicing of the green and amber 38 genes of the R39 panel. Even so, more than half of the families remain genetically unsolved, highlighting the limitation of this approach. Causative variants may lie in genes outside the panel, and some cases cannot be fully resolved (for instance, when only a VUS is found, or when only a single heterozygous pathogenic variant is detected in an autosomal recessive gene). Unlike previous studies that typically focused on cohorts ascertained through a single specialty, such as ophthalmology clinics, our use of the 100K Genomes Project enabled analysis of a more heterogeneous, undifferentiated population referred from across clinical genetics, neurology, and ophthalmology. This broader recruitment strategy may mitigate some forms of ascertainment bias but also introduces phenotypic variability that can challenge variant interpretation. Our analysis focused on an in silico gene panel approach, which prioritises coding and splice-site variants in phenotype-relevant genes. This strategy does not comprehensively interrogate non-coding regulatory regions, deep intronic variants, or structural variations. Non-coding variants have been implicated in neurodevelopmental disorders with ophthalmological features (including *RNU4-2* and *RNU6*-associated conditions), and approximately 60% of individuals with neurodevelopmental disorders remain undiagnosed after exome-based testing^21,22^. While such variants are likely to represent a small proportion of infantile nystagmus cases, future studies incorporating comprehensive non-coding genome analysis may further improve diagnostic yield for currently unexplained cases.

The use of WGS allowed us to identify pathogenic variants in 36 genes outside the panel, leading to diagnoses in 45 families that would have been missed by a panel-only approach. Many of these off-panel diagnoses were unexpected syndromic or atypical causes of IN/albinism (for example, variants in *SOX10*, *MITF*, *PAX3*, and *SRD5A3*), highlighting the clinical value of broad genomic analysis. Similarly, we identified dual genetic diagnoses in 4 individuals from 3 unrelated families, illustrating the complexity of some patients’ combined phenotypes. The co-occurrence of two independently segregating disease-causing alleles at two distinct loci has been increasingly reported in molecular genetics studies^23,24^, in part due to the expanded scope of WES/WGS in routine genetic testing. Such cases underscore the importance of careful clinical evaluation and variant interpretation to recognise all coexisting conditions rather than attributing a complex presentation to a single cause.

Our analysis focused on SNVs and small indels identified through the standard 100KGP bioinformatics pipeline. Structural variants, copy number variations, and deep intronic variants were not systematically evaluated in this study. While these variant classes are less common causes of infantile nystagmus and albinism, they may contribute to a subset of currently unexplained cases. Similarly, our panel-based approach did not comprehensively interrogate non-coding regulatory regions, which represent an emerging frontier in rare disease diagnosis. Future studies incorporating comprehensive structural variant calling and non-coding genome analysis may further improve diagnostic yield.

This study expands the genetic landscape and mutation spectrum of IN and albinism by identifying 103 different variants across 16 genes of the R39 panel. The majority of these variants were family-specific (private), although approximately one-quarter were found in multiple families. *TYR* variants were the most common, consistent with other studies showing *TYR* as the predominant gene in OCA1^18^. Similarly, the *TYR* “*cis*-YQ” p.[Ser192Tyr; Arg402Gln] haplotype was the most frequently identified pathogenic variant in this cohort, representing 36% (45/124) of all *TYR* disease-causing alleles. This is about double the previously ~19% reported in other *TYR*-associated OCA1 cohorts^25^. Since the *cis*-YQ haplotype comprises two common *TYR* variants (allele frequencies ~36% and ~29% in the European population), it is often overlooked in analysis pipelines due to its high frequency^26^. Nevertheless, there is evidence confirming that the *cis*-YQ combination is a hypomorphic, but disease-causing allele of *TYR* (associated with OCA1B)^25,26^. Our findings underscore its clinical importance: failing to recognize and report the *cis*-YQ allele could lead to missed or under-appreciated diagnoses of albinism.

We next explored genotype-phenotype correlation as permitted through the available clinical data. Interestingly, despite the known effects of many implicated genes on ocular development^27^, we did not observe consistent genotype–refractive error correlations beyond the association between *PAX6* variants and anisometropia. This likely reflects the variable depth and completeness of phenotypic data within the Genomics England research environment, where refractive error information may be inconsistently captured across records. The heterogeneity in data entry formats, clinical coding, and levels of ophthalmic detail, particularly in cases not recruited through specialist eye services, may obscure underlying genotype–phenotype patterns.

In conclusion, an in silico WGS panel approach proved to be a time-efficient first step for genetic diagnosis in infantile nystagmus and albinism, achieving a diagnostic yield of ~46%. However, this strategy has inherent limitations, as it excludes non-coding, structural, and off-panel variants. Our findings demonstrate that the current UK diagnostic mainstay, the R39 nystagmus gene panel, would not capture several of the relevant genetic aetiologies identified in this cohort, including syndromic and dual molecular diagnoses. Moreover, we highlight the importance of recognising common pathogenic variants such as the *TYR cis*-YQ haplotype, which may be filtered out by standard analytical pipelines due to their high allele frequency in European populations, despite established disease associations. Together, these results underscore the value of comprehensive genomic testing in the diagnostic pathway for IN and albinism, particularly in undifferentiated clinical settings. Recognising genotype–phenotype correlations, particularly where syndromic gene variants are involved, can guide clinical management by prompting targeted surveillance for associated systemic features.

## Methods

### Phenotype-driven cohort development

Genomics England has approval from the Health Research Authority Committee East of England – Cambridge South ((REC Ref 14/EE/1112). Informed consent was obtained from all participants. The study was conducted in accordance with the ethical standards as outlined in the Declaration of Helsinki.

We used the Genomics England (GE) *Participant Explorer* tool to identify participants by relevant clinical terms. The search logic ‘Has Any Of’ was applied to include any participant with at least one of the clinical terms of interest. We queried three coding systems for phenotype terms: Human Phenotype Ontology (HPO), International Classification of Diseases 10th Revision (ICD-10) and the GE Rare Diseases taxonomy (a three-level coding systems of rare disease groups, subgroups and specific diseases used in 100KGP). The pre-defined list of terms included HPO entries (with their descendant terms) for “Albinism” (HP:0001022), “Partial albinism” (HP:0007443), “Ocular Albinism” (HP:0001107), “Congenital nystagmus” (HP:0006934, HP:0007859, HP:0007811) and “Aplasia/Hypoplasia of the fovea” (HP:0008060, HP:0011503, HP:0007750). In addition, we included the ICD-10 code for Albinism (E70.3, 2016 version) and the GE rare disease code for Infantile nystagmus.

LabKey within the GE research environment is a data server containing all the clinical and phenotypic data of the participants in addition to the results of bioinformatics analysis. The LabKey client libraries (APIs) provide programmatic access to LabKey data where data were visualized and analysed using *R* version 4.0.2 (2020-06-22) through Rstudio 2022.12.0 build 353. Extended clinical, phenotypic, demographic and family data of each participant were extracted from the GE Main Programme Release 19 (31 October 2024).

### Clinical features enrichment analysis

We performed enrichment analysis for clinical features in our phenotype-defined cohort versus the broader 100KGP rare disease dataset. For each coding system (HPO, ICD-10 and GE Rare Diseases), we used a hypergeometric function phyper() in *R* to identify terms significantly over-represented in our cohort. *P*-values were corrected for multiple testing using the False Discovery Rate method (p.adjust() in R). We also calculated the odds ratio for cohort inclusion associated with each enriched feature, to quantify the enrichment effect.

### Genetic data analysis workflow

We analysed genomic data from WGS focusing on an in silico targeted gene panel and included patients solved through Genomic Medicine Centre (GMC) laboratories by variants found in other genes outside the gene panel.

We applied the GE PanelApp gene panel “R39 Albinism or congenital nystagmus – V3.0” (NHS Genomic Test Directory) as our primary gene list. In PanelApp, genes are tagged Green (diagnostic-grade evidence), Amber (moderate evidence) or Red (limited evidence). We included variants in Green or Amber genes from the R39 panel for initial analysis.

In the GE Rare Diseases data processing pipeline, coding variants (single nucleotide variants (SNVs) and indels) are automatically stratified into Tier 1, 2 or 3 based on their segregation, population frequency, predicted impact on protein coding, mode of inheritance and presence in the phenotype-driven gene panel(s) for each case. We utilised this tiering: Tier 1 variants (high impact variants or de novo moderate impact variants in Green panel genes) and Tier 2 variants (moderate impact variants in Green panel genes). Tier 3 variants (potentially relevant high and moderate impact variants in genes outside the curated list) were also reviewed for broader diagnoses. We extracted all tiered variants in R39 panel genes for each affected participant and their family members.

For participants with no pathogenic/likely pathogenic Tier 1 or 2 variants, we performed a re-analysis of their WGS data. We extracted all coding and splice-region variants ( ± 25 bp from exons) in R39 panel genes from the variant call files (VCFs) of these participants and their relatives. To ensure consistency, any VCF data aligned to GRCh37 was lifted over to GRCh38. We annotated all candidate variants using the Ensembl Variant Effect Predictor (VEP v111). We then applied filters to prioritise variants: we removed variants above population frequency of 0.01 (using gnomAD), we assessed predicted pathogenicity using in silico tools (PolyPhen-2, SIFT, CADD (only variants with a PHRED score of more than 20 were prioritized and SpliceAI (variants with a score more than 0.6 were prioritized). Variants classified as benign or likely benign in ClinVar were excluded. The remaining variants were interpreted and assigned pathogenicity based on American College of Medical Genetics and Genomics (ACMG) guidelines^28^.

We implemented a custom step to detect the *TYR* “*cis*-YQ” haplotype p.[Ser192Tyr;Arg402Gln], which is a known pathogenic low-penetrance allele in OCA1B^25^. The two constituent variants are common in the population, so they were not flagged by routine tiering or our frequency filters. We specifically queried all unsolved cases for these two variants. Participants homozygous for both variants were considered homozygous for *cis*-YQ haplotype; if one variant was homozygous and the other heterozygous, they were considered obligate carrier of the *cis*-YQ haplotype. We then examined segregation within families to confirm inheritance of the *cis*-YQ allele.

We reviewed the accredited GMC reports (exit questionnaire) for each family. These reports indicate whether a genetic cause was found that fully or partially explains the participant’s condition or no genetic diagnosis was reached. We retrieved the outcome for 384 of the 388 families (for 4 families, no GMC outcome data were available). Recruiting clinicians were contacted for further clinical findings and communicating potential genetic diagnosis through the GE clinical collaboration request system.

## Supplementary information

Supplementary Data.

Supplementary information.

## Data Availability

Data from the 100kGP and the Genomic Medicine Service are held in the National Genomic Research Library, Genomics England, 10.6084/m9.figshare.4530893.v8. Research on the de-identified patient data used in this publication can be carried out in the Genomics England Research Environment subject to a collaborative agreement that adheres to patient led governance. All interested readers will be able to access the data in the same manner that the authors accessed the data. For more information about accessing the data, interested readers may contact research-network@genomicsengland.co.uk or access the relevant information on the Genomics England website: https://www.genomicsengland.co.uk/research.
