# Supplementary material for: Whole-genome sequencing uncovers diverse genetic causes and phenotypic signatures in infantile nystagmus and albinism
Source: NPJ Genom Med. 2026 May 20;11:43. doi: 10.1038/s41525-026-00580-1 (PMC13454288; doi:10.1038/s41525-026-00580-1)
Supplement: Supplementary file 1 [file 41525_2026_580_MOESM1_ESM.pdf]

## Supplementary Information

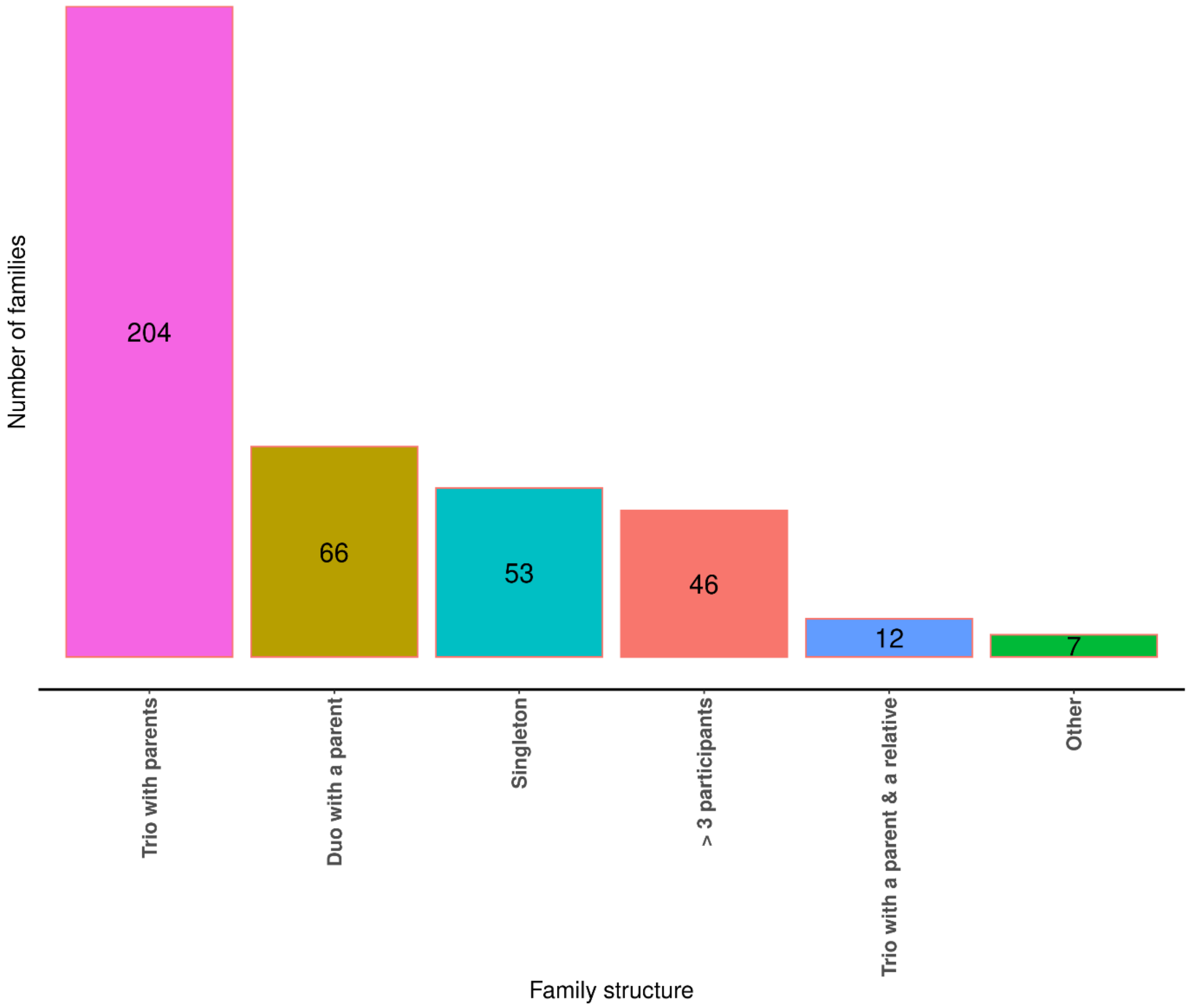


**Figure S1: Bar chart of the number of family structures included in the studied cohort.** Over half of the families were trio with parents.

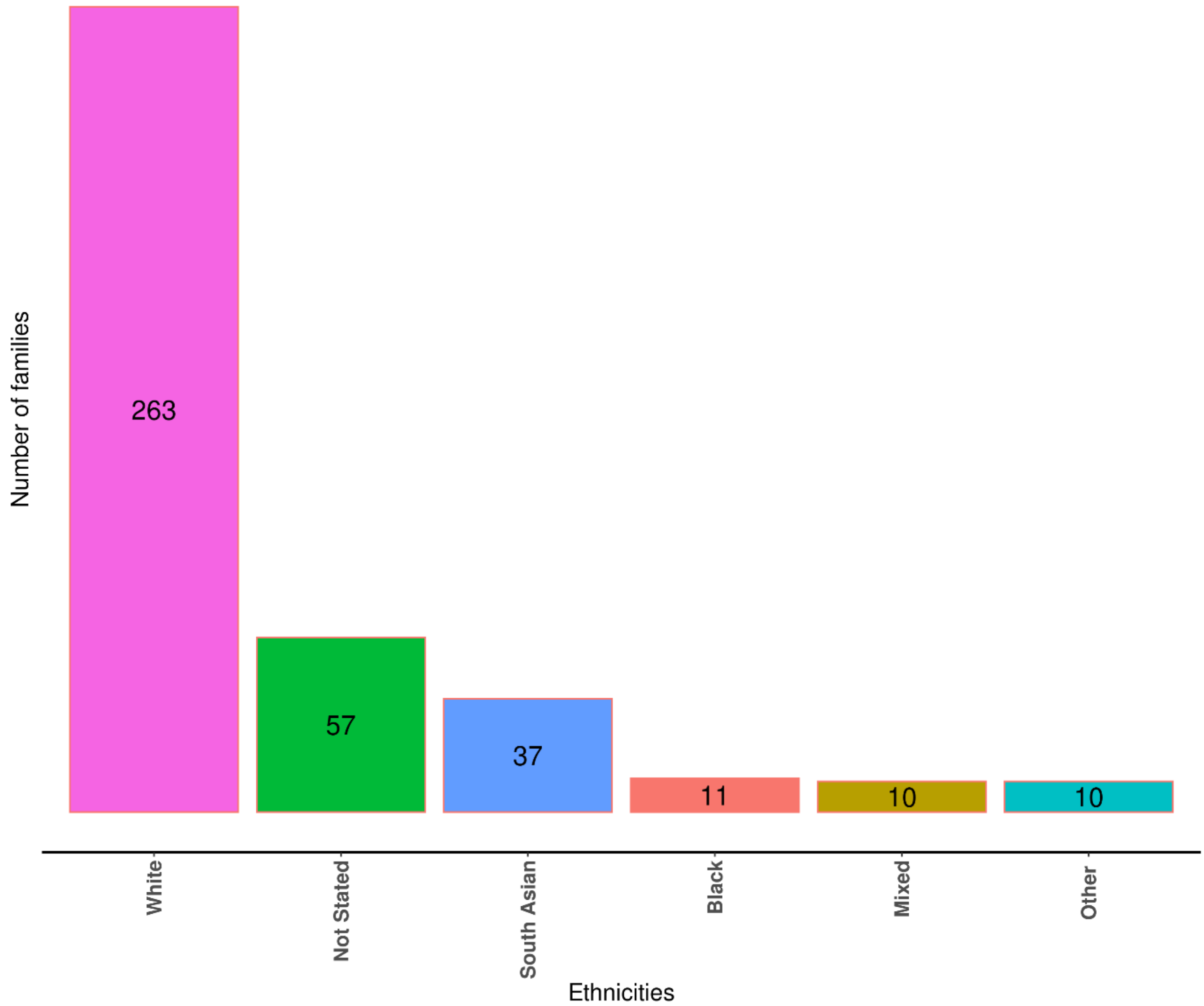


**Figure S2: Bar chart of the number of families in various ethnic groups.** Most of the families were White Caucasians

**Supplementary Data**

Supplementary Data1: **A list of HPO terms used to describe participants phenotypes and their frequency**

Supplementary Data2: **A list of ICD-10 terms used to describe participants phenotypes and their frequency**

Supplementary Data3: **A list of GE Disease Groups used to describe participants phenotypes and their frequency**

Supplementary Data4: **A list of GE specific diseases used to describe participants phenotypes and their frequency**

Supplementary Data5: **Enrichment of HPO terms within the infantile nystagmus/albinism cohort**

Supplementary Data6: **Enrichment of ICD-10 terms within the infantile nystagmus/albinism cohort**

Supplementary Data7: **Enrichment of GE specific diseases within the infantile nystagmus/albinism cohort**

Supplementary Data8: **A list of identified variants in R39 panel genes**

Supplementary Data9: **A list of variants identified by GMC reports in genes outside the R39 panel**
